# Decoding endurance performance: integrative genomic and epigenetic insights into the molecular basis of athletic adaptation

**DOI:** 10.3389/fsysb.2026.1858520

**Published:** 2026-09-25

**Authors:** Arij El Haddouchi, Amina Marrouh, Souad Kartti, Elmostafa El Fahime, Saber Boutayeb, Younes Chagar, Christophe Baudot, Lahcen Belyamani, Rachid Eljaoudi, Taoufiq Dakka

**Affiliations:** 1 Interdisciplinary Laboratory of Biotechnology and Health, Mohammed VI Higher Institute of Biosciences and Biotechnology, Mohammed VI University of Sciences and Health (UM6SS), Casablanca, Morocco; 2 Mohammed VI University of Sciences and Health, Morocco - UM6SS, Casablanca, Morocco; 3 Mohammed VI Center for Research and Innovation (CM6RI), Rabat, Morocco; 4 Laboratory of Drugs Science Research, Mohammed VI Faculty of Pharmacy, Mohammed VI University of Sciences and Health (UM6SS), Casablanca, Morocco; 5 Sports and Health Unit, Mohammed VI Foundation of Science and Health (FM6SS), Rabat, Morocco; 6 Rabat Medical and Pharmacy School, Mohammed V University, Rabat, Morocco

**Keywords:** athletes, endurance athlete status, epigenetics, genetic variants, MicroRNAs

## Abstract

Applied genomics in sports performance has rapidly evolved into a multidisciplinary field aimed at elucidating the biological determinants of complex athletic traits, particularly endurance. Endurance performance represents a highly polygenic and multifactorial phenotype, shaped by the dynamic interplay between genetic predisposition and training-induced adaptations. This review provides a comprehensive synthesis of evidence from candidate gene studies, genome-wide association studies (GWAS), and meta-analyses to identify genetic variants consistently associated with endurance-related phenotypes. GWAS and candidate gene approaches have identified over 44 polymorphisms relevant to endurance-oriented sports, with *HFE* rs1799945 emerging as a strong predictor of endurance ability. Key variants, including *ACE* rs1799752, *CDKN1A* rs236448, *PPARGC1A* rs8192678, and *PPARA* rs4253778, are highlighted for their roles in modulating critical physiological pathways such as oxygen transport, mitochondrial biogenesis, energy metabolism, and muscle fiber composition. Beyond static genetic variation, this review emphasizes the pivotal contribution of epigenetic mechanisms, including DNA methylation, histone modifications, and non-coding RNAs. In particular, exercise-responsive microRNAs (miR-1, miR-206, miR-21, miR-146, miR-208, and miR-222) are discussed as key regulators of gene expression and molecular adaptation to endurance training. Although more than 200 genetic polymorphisms have been associated with athlete status, only a limited subset demonstrates consistent and replicated associations with endurance athlete status. This underscores the limitations of single-marker approaches and highlights the need for integrative frameworks that incorporate genomics, epigenomics, and transcriptomics. Advancing toward such multi-omics strategies is essential for a more comprehensive understanding of the biological architecture of endurance, and for the development of personalized training interventions aimed at optimizing performance and adaptation.

## 1 Introduction

Over the past 2 decades, the investigation of endurance performance has evolved from a reductionist focus on single genetic variants toward a more integrative and dynamic understanding of human physiological adaptation (Bıçakçı et al., 2024; Yao et al., 2025). Endurance, as a complex and highly plastic phenotype, emerges from the interplay between genetic predisposition, molecular regulation, and environmental stimuli (Bıçakçı et al., 2024; Georgiades et al., 2017). While early research emphasized the contribution of specific polymorphisms to athletic status, recent advances have revealed that endurance capacity is underpinned by a highly polygenic architecture, in which numerous genetic variants exert small but cumulative effects across interconnected biological pathways (Bıçakçı et al., 2024). This polygenic nature presents a major challenge for sports genomics research, as no single variant is sufficient to explain endurance elite status. Rather, athletic success likely results from the combined influence of multiple loci interacting with environmental, physiological, and training-related factors. Consequently, identifying an optimal combination of endurance-associated variants remains difficult, particularly in elite athlete populations where recruitment opportunities are inherently limited. These challenges underscore the need to complement genetic association studies with deeper investigations of the molecular, biochemical, and regulatory mechanisms underlying endurance adaptations (Williams and Folland, 2008). Despite the identification of well-characterized candidate genes such as *ACE* (Jones et al., 2002), *ACTN3* (Yang et al., 2003), and *PPARGC1A* (Lucia et al., 2005), the explanatory power of genetic variation alone remains limited. Indeed, inconsistencies across populations and the presence of elite athletes lacking “favorable” genotypes highlight a critical gap in the deterministic interpretation of sports genomics. This discrepancy has prompted a conceptual shift toward models that incorporate regulatory layers beyond DNA sequence variation, particularly those governing gene expression dynamics (Mallett, 2025; Barrès et al., 2012; Wardle et al., 2015; Baggish et al., 2011).

In this context, epigenetic mechanisms have emerged as key modulators of exercise-induced adaptations. Processes such as DNA methylation, histone modifications, and non-coding RNA activity, especially microRNAs, enable rapid and reversible changes in gene expression in response to environmental stimuli, including physical training (Mallett, 2025; Geiger et al., 2024; Jacques et al., 2025; Hiam et al., 2023). These mechanisms play a central role in regulating pathways related to mitochondrial biogenesis, oxidative metabolism, inflammation, and muscle remodeling, thereby contributing to both acute responses and long-term adaptations to endurance exercise (Clauss et al., 2016; Baggish et al., 2014). Unlike static genetic variants, epigenetic modifications provide a flexible interface through which environmental factors can shape the functional output of the genome (Jacqu et al., 2019). Importantly, this epigenetic plasticity offers a compelling explanation for the marked inter-individual variability observed in training responsiveness. While some individuals exhibit substantial improvements in aerobic capacity (“high responders”), others show limited adaptation despite similar training stimuli (El Haddouchi et al., 2025; Mann et al., 2014; Marrouh et al., 2025; Jacques et al., 2023). This phenomenon suggests that endurance performance is not solely determined by inherited genetic variants, but also by the capacity of the epigenome to dynamically regulate gene expression in response to physiological stress (Bıçakçı et al., 2024). Consequently, the interaction between genotype and epigenetic regulation represents a critical, yet still underexplored, dimension of athletic performance (Jacqu et al., 2019).

Furthermore, most genomic studies in sports science have been conducted in relatively homogeneous populations, limiting the generalizability of findings and potentially overlooking population-specific genetic and epigenetic signatures. Addressing this gap is essential for developing a more comprehensive and inclusive understanding of endurance biology, particularly in underrepresented populations where unique gene-environment interactions may exist. Therefore, advancing the field requires moving beyond static genetic associations toward integrative frameworks that combine genomics, systems physiology, epigenomics, transcriptional responses, training exposure, and environmental factors interact to shape endurance-related phenotypes (Figure 1). Such an approach is crucial not only for elucidating the biological foundations of endurance performance but also for enabling future applications in personalized training, performance optimization, and injury prevention, while carefully considering ethical implications. In this context, the present review aims to provide a comprehensive and updated synthesis of both genetic and epigenetic determinants of endurance performance, with a particular focus on key polymorphisms, regulatory mechanisms such as histone modifications and microRNA dynamics, and their combined role in shaping inter-individual variability in training adaptation.

**FIGURE 1:**
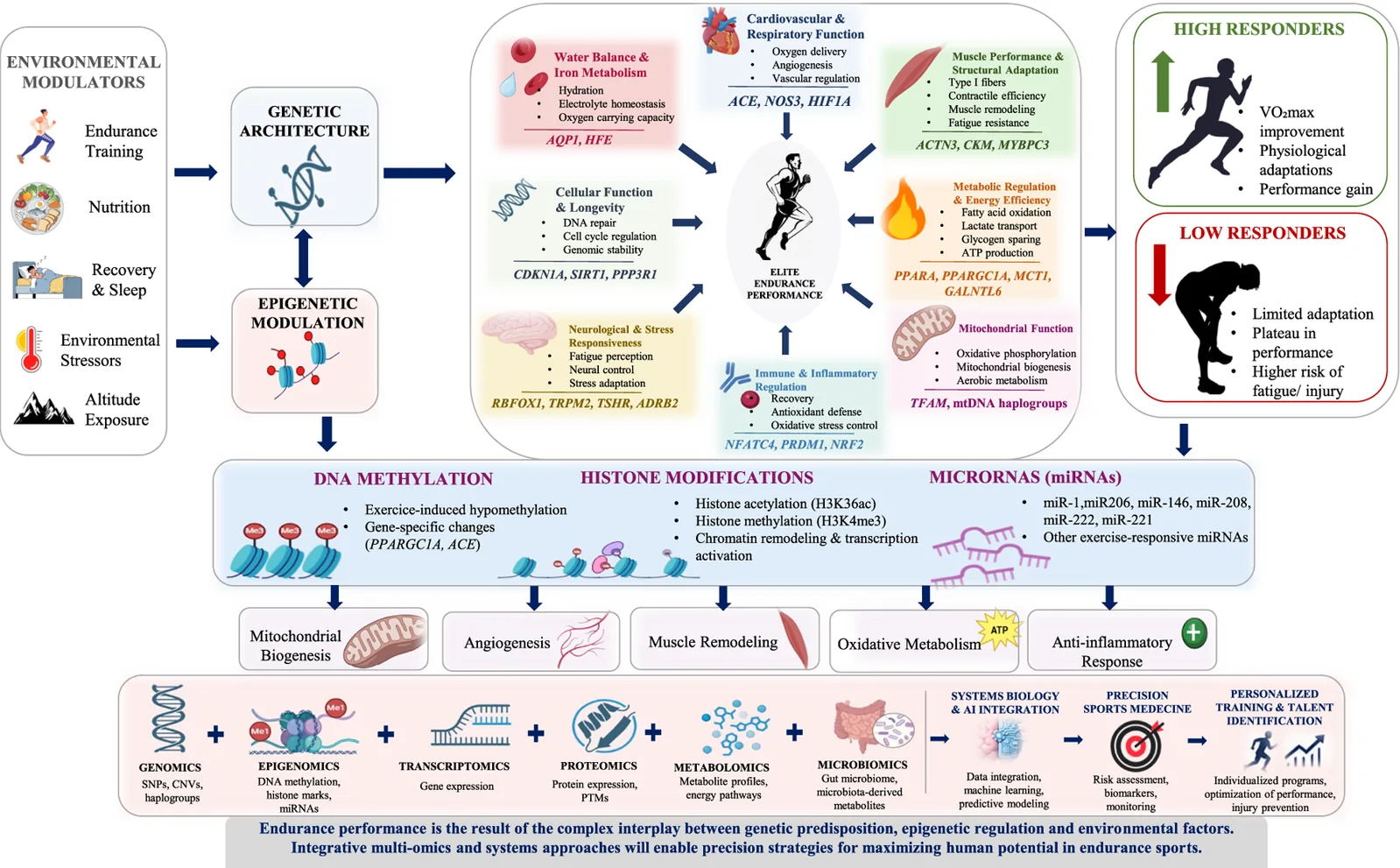
Integrated genetic, epigenetic, and environmental determinants of endurance athlete status and training responsiveness.

## 2 Literature search strategy

This narrative review was conducted using a structured and comprehensive literature search strategy to identify studies investigating the genetic and epigenetic determinants of endurance performance and training adaptation in athletes. Electronic searches were performed in PubMed, Scopus, Web of Science, and Google Scholar to retrieve relevant articles published up to April 2026. The search strategy combined Medical Subject Headings (MeSH) and free-text keywords related to endurance performance, exercise genetics, and exercise epigenetics, including “endurance athlete”, “endurance performance”, “aerobic capacity”, “VO_2_max”, “cardiorespiratory fitness”, “exercise adaptation”, “genetics”, “genetic polymorphism”, “single nucleotide polymorphism”, “SNP”, “gene variant”, “genome-wide association study”, “GWAS”, “epigenetics”, “DNA methylation”, “histone modification”, “microRNA”, and “exercise-responsive genes”.

For the purpose of this review, endurance sports were defined as sports in which performance predominantly depends on the capacity to sustain prolonged aerobic energy production and oxygen utilization. These included long-distance running, road cycling, swimming, rowing, cross-country skiing, triathlon, and other disciplines characterized by sustained aerobic exercise. As no generally applicable distance threshold can be applied across different endurance disciplines, classification was based primarily on the predominant contribution of aerobic metabolism and the sustained nature of the exercise rather than on a specific distance criterion.

Studies were considered eligible if they: (Bıçakçı et al., 2024) investigated genetic variants, epigenetic mechanisms, or their interactions in relation to endurance performance, aerobic fitness, training responsiveness, or physiological adaptations to endurance exercise; (Yao et al., 2025) included participants engaged in endurance sports, including elite, professional, semi-professional, or recreational athletes; and (Georgiades et al., 2017) were published in peer-reviewed journals in English. Candidate-gene association studies, genome-wide association studies (GWAS), epigenetic investigations, systematic reviews, and meta-analyses providing relevant evidence were considered. Studies focusing exclusively on non-exercise-related phenotypes, animal models without clear translational relevance, conference abstracts lacking sufficient methodological details, and publications unavailable in full text were excluded.

The selection process involved the evaluation of titles, abstracts, and full texts according to their relevance to the objectives of the review. Information extracted from eligible studies included the investigated genes, polymorphisms or epigenetic markers, study design, participant characteristics, sport discipline, endurance-related phenotypes, and the reported associations with performance or training adaptation. To facilitate interpretation, the identified genetic and epigenetic factors were subsequently organized according to their predominant biological functions, including cardiovascular regulation, skeletal muscle structure and function, energy metabolism, mitochondrial biogenesis, oxygen transport, angiogenesis, immune and inflammatory responses, oxidative stress regulation, and epigenetic control of exercise adaptation.

## 3 Genetics and endurance athlete status

Endurance performance is fundamentally rooted in the capacity to sustain aerobic energy production over prolonged periods, a process primarily driven by mitochondrial efficiency and oxygen utilization. At the cellular level, mitochondria orchestrate oxidative phosphorylation, ensuring continuous ATP supply required for repeated muscle contractions (Lippi et al., 2010). In elite endurance athletes, enhanced mitochondrial density and function, often accompanied by coordinated upregulation of genes involved in oxidative metabolism, constitute a central hallmark of superior physiological capacity (Bıçakçı et al., 2024; Maruszak et al., 2014). However, endurance is not governed by a single biological system, but rather emerges from the integration of multiple physiological components. These include a high proportion of type I (slow-twitch) muscle fibers, optimized cardiovascular function characterized by elevated cardiac output, and efficient oxygen transport and extraction (Ahmetov et al., 2022). Collectively, these factors converge to determine maximal oxygen uptake (VO_2_max), an important indicator of aerobic capacity and endurance performance. In parallel, endurance training induces metabolic adaptations such as increased lipid oxidation, improved glycogen sparing, and enhanced lactate clearance, all of which contribute to delaying fatigue and sustaining performance (Ahmetov et al., 2022; Hellsten and Nyberg, 2015; Bergh et al., 1978).

Within this multifactorial framework, genetic factors play a substantial role in shaping inter-individual variability in endurance-related phenotypes. Heritability estimates suggest that up to 44%–68% of the variance in VO_2_max and aerobic capacity can be attributed to genetic influences (Miyamoto-Mikami et al., 2018). Nevertheless, this contribution must be interpreted within a broader context, as environmental factors, including training load, nutrition, and lifestyle, interact dynamically with genetic predisposition (Bıçakçı et al., 2024).

Traditionally, the identification of genetic determinants of endurance has relied on case-control designs comparing elite athletes with non-athletic populations, leading to the discovery of numerous candidate polymorphisms associated with endurance status (Semenova et al., 2023). Importantly, these candidate gene and SNP-based association studies have played a pivotal role in advancing sports genomics, particularly given the rarity of elite athlete cohorts and the considerable challenges associated with accessing highly specialized athletic populations. They provided the first evidence linking genetic variation to endurance-related phenotypes and established the foundation for subsequent genome-wide and multi-omics investigations. While this approach has generated valuable insights, it remains limited in its ability to fully capture the complexity of genotype-phenotype relationships. In particular, the functional mechanisms through which these variants influence physiological adaptation, as well as their interaction with regulatory processes such as epigenetic modifications, are still insufficiently understood (Bıçakçı et al., 2024; Varley et al., 2018; Williams et al., 2025).

Consequently, a major challenge in the field is to move beyond associative studies toward integrative models that link genetic variation to biological function and training responsiveness. Addressing this gap is essential for advancing our understanding of how genetic architecture contributes not only to baseline endurance capacity but also to the adaptive processes that ultimately define elite performance.

The genetic architecture of endurance performance encompasses variants involved in multiple interconnected physiological systems, reflecting the multifactorial nature of elite athletic performance. Candidate gene studies have identified polymorphisms associated with cardiovascular and respiratory function, muscle performance, metabolic efficiency, immune and inflammatory regulation, neurological responses, cellular maintenance, fluid homeostasis, and iron metabolism. Although the contribution of individual variants is generally modest, their combined effects may influence endurance-related phenotypes and the likelihood of attaining elite athlete status. Table 1 summarizes the principal genetic variants associated with endurance performance according to their predominant biological functions.

**TABLE 1:** Key genetic variants associated with endurance athlete status and their biological roles.

Gene	Gene name	Key SNPs	Chromosomal position	Endurance associated allele, (References)
*ACE*	Angiotensin I converting enzyme	rs1799752 I/D	17q23.3	I, (Gayagay et al., 1998)
*AGTR2*	Angiotensin II receptor type 2	rs11091046A > C	Xq23	C, (Mustafina et al., 2014)
*BDKRB2*	Bradykinin receptor B2	rs5810761 + 9/-9 (exon 1)	14q32.2	− 9, (Williams et al., 2003)
*HIF1A*	Hypoxia inducible factor 1 alpha subunit	rs11549465 C/T	14q23.2	C, (Prior et al., 2003)
*NOS3*	Nitric oxide synthase 3	rs2070744 T>C	7q36.1	T, (Zmijewski et al., 2018)
*VEGFA*	Vascular endothelial growth factor A	rs2010963 G>C	6p21.1	C, (Ahmetov et al., 2009a)
*VEGFR2*	Vascular endothelial growth factor receptor 2	rs1870377 T/A	4q12	A, (Ahmetov et al., 2009b)
*EGLN1*	Egl-9 family hypoxia-inducible factor 1	rs1614148 C>A	1q42.2	A, (Dautova et al., 2024)
*ACTN3*	Actinin alpha 3	rs1815739 C>T	11q13.2	T, (Yang et al., 2003)
*CKM*	Creatine kinase M-type	rs8111989A > G	19q13.32	A, (Zhou et al., 2006)
*MYBPC3*	Myosin binding protein C3	rs1052373A > G	11p11.2	G, (Al-Khelaifi et al., 2020)
*SPEG*	Striated muscle enriched protein kinase	rs7564856 G>A	2q35	G, (Kusić et al., 2020)
*COL5A1*	Collagen type V alpha 1 chain	rs12722 C>T	9q34.3	T, (Posthumus et al., 2011) (Brown et al., 2011)
*AMPD1*	Adenosine monophosphate deaminase 1	1p13	rs17602729 C/T	C, (Kartibou et al., 2025)
*PPARA*	Peroxisome proliferator-activated receptor alpha	rs4253778 G>C	22q13.31	G, (Ahmetov et al., 2006)
*PPARGC1A*	Peroxisome proliferator-activated receptor, gamma, coactivator 1 alpha	rs8192678 G>A	4p15.2	G, (Ahmetov et al., 2009a)
*PARGC1B*	Peroxisome proliferator-activated receptor, gamma, coactivator 1 beta	rs7732671 G>C	5q32	C, (Akhmetov et al., 2009)
*UCP2*	Uncoupling protein 2	rs660339 C>T	11q13.4	T, (Ahmetov et al., 2008)
*UCP3*	Uncoupling protein 3	rs1800849 C>T	11q13.4	T, (Ahmetov et al., 2009a)
*FTO*	FTO alpha-ketoglutarate dependent dioxygenase	rs9939609 T>A	16q12.2	T, (Zmijewski and Leońska-Duniec, 2021)
*GALNTL6*	Polypeptide N-acetylgalactosaminyltransferase 6	rs558129 T>C	4q34.1	C, (Rankinen et al., 2016)
*MtDNA loci*	Mitochondrial DNA	MtDNA haplogroups	MtDNA	H, NA (Maruszak et al., 2014)
				Unfavourable: K, NA (Maruszak et al., 2014)
*TFAM*	Transcription factor A, mitochondrial	rs1937 G>C	10q21.1	C, (Ahmetov et al., 2009a)
*MCT1*	Monocarboxylate transporter 1	rs1049434A > T	1p13.2	T, (Guilherme et al., 2021)
*NFATC4*	Nuclear factor of activated T cells 4	rs2229309 G>C	14q12	G, (Ahmetov et al., 2009a)
*PRDM1*	PR/SET Domain 1	rs10499043 C/T	6q21	T, (Hall et al., 2021a)
*NRF2*	Nuclear respiratory factor	rs12594956 A/C	2q31.2	A, (Eynon et al., 2010a)
*IL6*	Interleukin-6	rs1800795 −174G/C	7p15.3	C, (Ben-Zaken et al., 2017)
*RBFOX1*	RNA binding fox-1 homolog 1	rs7191721 G>A	16p13.3	G, (Ahmetov et al., 2015)
*TRPM2*	Transient Receptor Potential Cation Channel Subfamily M Member 2	rs1785440 A/G	21q22.3	G, (Bulgay et al., 2023a)
*ADRB2*	Adrenoceptor beta 2	rs1042713 G>A	5q32	A, (Wolfarth et al., 2007)
		rs1042714 G/C		C, (Varillas-Delgado et al., 2022)
*ADRB3*	Adrenoceptor beta 3	rs4994 A/G	8p11.23	G, (Santiago et al., 2011)
*TSHR*	Thyroid stimulating hormone receptor	rs7144481 T>C	14q31.1	C, (Ahmetov et al., 2015)
*CDKN1A*	Cyclin-dependent kinase inhibitor 1A	rs236448 A/C	6p21.2	A, (Semenova et al., 2022)
*NUP210*	Nucleoporin 210	rs2280084	3p25.1	A, (Bulgay et al., 2023a)
*SIRT*	Sirtuin 1	rs41299232 C/G	10q21.3	G, (Bulgay et al., 2023a)
*GABPB1*	GA binding protein transcription factor subunit beta 1	rs7181866A > G	15q21.2	G, (He et al., 2007; Eynon et al., 2009)
*NFIA-AS2*	NFIA antisense RNA 2	rs1572312 C>A	1p31.3	C, (Ahmetov et al., 2015)
*PPP3R1*	Protein phosphatase 3 regulatory subunit B, alpha	Promoter 5I/5D	2p14	5I, (Ahmetov et al., 2009a)
*AQP1*	Aquaporin 1	rs1049305 C > G	7p14.3	C, (Rivera et al., 2020)
*HFE*	Homeostatic iron regulator	rs1799945	6p21.3	G, (Semenova et al., 2020)

### 3.1 Genetic determinants of cardiovascular and respiratory function in endurance athletes

Cardiovascular and respiratory systems constitute the central axis of endurance performance, as they collectively determine the efficiency of oxygen delivery, utilization, and metabolic waste removal during prolonged exercise (Gayagay et al., 1998; Prior et al., 2003; Hughes et al., 2018). Rather than acting independently, these systems operate as an integrated network in which cardiac output, vascular function, and pulmonary gas exchange are tightly coordinated to meet the energetic demands of sustained muscular activity (Green et al., 2012; Döring et al., 2010). At the physiological level, endurance capacity relies on the ability to maximize oxygen transport from the atmosphere to the mitochondria, where it is used for ATP production (Gayagay et al., 1998). This process is supported by structural and functional adaptations such as increased stroke volume, enhanced capillary density, and improved pulmonary diffusion capacity. In parallel, regulatory mechanisms controlling heart rate, vascular tone, and blood pressure play a critical role in optimizing performance and accelerating post-exercise recovery (Green et al., 2012; Döring et al., 2010; Qian et al., 2019).

Importantly, inter-individual variability in these traits is partially driven by genetic factors that influence cardiovascular regulation, vascular responsiveness, and oxygen transport efficiency (Zmijewski et al., 2018; Wei, 2021).

#### 3.1.1 Genetic regulation of vascular function and blood pressure

Genes involved in vascular homeostasis and blood pressure regulation have been extensively investigated in relation to endurance performance, primarily due to their role in modulating blood flow and tissue oxygenation (Gayagay et al., 1998; Mustafina et al., 2014).

Among these, the *ACE* gene remains one of the most extensively studied. The insertion (I) allele of the rs1799752 polymorphism has frequently been associated with elite endurance athlete status and higher VO_2_max responses to endurance training, potentially through reduced angiotensin-converting enzyme activity, leading to improved vasodilation and metabolic efficiency (Gayagay et al., 1998; Ribas et al., 2023). However, findings across studies remain heterogeneous, with several investigations failing to replicate this association, suggesting that its effect may be modulated by population-specific factors or gene-environment interactions (Amir et al., 2007; Saito et al., 2022). Furthermore, recent evidence indicates that the *ACE* II genotype alone may not be strongly associated with endurance athlete status. Instead, its combined effect with the X allele of the rs1815739 polymorphism in the *ACTN3* gene (RX or XX genotypes) appears to significantly increase the likelihood of being an endurance athlete, highlighting the importance of gene-gene interactions in shaping endurance-related phenotypes (Chae et al., 2024). Furthermore, one study conducted in untrained, normotensive young males from Southeast Asia reported that the ACE II genotype was associated with an enhanced response to low-intensity endurance training, resulting in a significant improvement in aerobic performance after 8 weeks (p = 0.0355) (Jones et al., 2016). Similarly, the *AGTR2* gene, which encodes the angiotensin II receptor type 2, has been implicated in cardiovascular adaptation. The rs11091046 polymorphism has shown associations with endurance athlete status, with the C allele being more prevalent in endurance athletes compared to strength athletes and non-athletes in certain cohorts (Mustafina et al., 2014). Nevertheless, conflicting evidence from subsequent studies underscores the complexity of its functional impact and limits definitive conclusions (Guilherme et al., 2018). Vascular function is also strongly influenced by nitric oxide-mediated signaling pathways, in which the *NOS3* gene plays a central role. The -786T/C (rs2070744) polymorphism has been associated with endurance performance, with the TT genotype linked to enhanced nitric oxide production and improved vasodilation. This genotype appears to be overrepresented in endurance athletes, particularly in disciplines requiring sustained aerobic output such as long-distance swimming (Zmijewski et al., 2018). Another relevant gene is *BDKRB2*, which encodes the bradykinin B2 receptor and contributes to vasodilatory responses. The −9/+9 insertion/deletion polymorphism has been associated with endurance traits, with the −9/-9 genotype potentially enhancing receptor expression and metabolic efficiency (Williams et al., 2003; Saunders et al., 2006a; Saunders et al., 2006b). While some studies report an overrepresentation of this genotype in elite endurance athletes, others have found no significant associations, highlighting the variability and context-dependency of these genetic effects (Eynon et al., 2011; Grenda et al., 2014; Sawczuk et al., 2013). Collectively, these findings suggest that while vascular-related genes contribute to endurance performance through modulation of blood flow and oxygen delivery, their individual effects are modest and often influenced by complex interactions with other genetic and environmental factors.

#### 3.1.2 Genetic influences on oxygen transport and utilization

Efficient oxygen transport and utilization represent a critical determinant of endurance performance, linking cardiovascular function to cellular energy production. Genetic variants involved in hypoxia response, angiogenesis, and muscle oxygen extraction have therefore attracted considerable attention (Döring et al., 2010; Jerzy et al., 2013). The *HIF1A* gene, a key regulator of cellular adaptation to hypoxia, has been widely studied in this context. Variants such as Pro582Ser (C1772T) have been associated with improved aerobic training responsiveness and maintenance of high VO_2_max levels (Prior et al., 2003; Döring et al., 2010). Angiogenesis-related genes also play a pivotal role in endurance adaptation. The *VEGFA* gene, which regulates vascular endothelial growth, has been associated with improved aerobic capacity through enhanced capillarization and oxygen delivery to skeletal muscle. Specifically, the C allele of the rs2010963 polymorphism has been linked to enhanced aerobic capacity and increased maximal energy production (Ahmetov et al., 2009a; Akhmetov et al., 2008; Prior et al., 2006). In parallel, the *VEGFR2* gene, encoding a major receptor for VEGF signaling, contributes to the regulation of vascular remodeling in response to training. The His472Gln polymorphism has shown strong associations with endurance athlete status, with the Gln allele being more frequent in endurance athletes, particularly in sports requiring sustained aerobic output such as rowing and long-distance swimming. This variant has been linked to improved oxygen uptake and a higher proportion of oxidative muscle fibers, further supporting its role in endurance phenotypes (Ahmetov et al., 2009b; Jerzy et al., 2013). More recently, the *EGLN1* gene, involved in hypoxia sensing through regulation of the HIF pathway, has emerged as a relevant contributor. The rs1614148 polymorphism has been associated with endurance status, with the A allele more frequent in endurance athletes than in power athletes (26.3% vs. 15.8%, OR = 1.90, p = 0.025). In addition, the AA genotype has been linked to higher VO_2_max values among top-level endurance athletes (International Master of Sports and Honored Master of Sports) (p = 0.047) (Dautova et al., 2024).

### 3.2 Genetic determinants of muscle performance and structural adaptation

Skeletal muscle represents the primary effector tissue underlying endurance performance, integrating metabolic, structural, and contractile functions to sustain prolonged physical activity. Rather than being a static entity, muscle tissue exhibits remarkable plasticity, adapting to both genetic predisposition and environmental stimuli such as training (Furrer et al., 2023; Furrer and Handschin, 2024; Smith et al., 2023). At the functional level, endurance capacity is strongly influenced by muscle fiber composition (Hall et al., 2021b). Type I (slow-twitch) fibers, characterized by high oxidative capacity, mitochondrial density, and fatigue resistance, are preferentially associated with endurance performance (Bergh et al., 1978). In contrast, type II (fast-twitch) fibers are specialized for rapid force production and anaerobic metabolism, favoring power and sprint activities (El Haddouchi et al., 2025; Hall et al., 2021b). Beyond fiber type distribution, additional factors such as calcium handling, ATP turnover efficiency, and fiber hypertrophy contribute to the overall contractile and metabolic performance of skeletal muscle (Kusić et al., 2020; Lai et al., 2008). Genetic variation plays a central role in modulating these muscular properties, influencing not only fiber composition but also structural integrity and adaptive potential (Zhou et al., 2006; Pickering and Kiely, 2017).

#### 3.2.1 Genetic influences on muscle fiber composition and contractile function

Among the most extensively studied genes in muscle physiology, *ACTN3* has been widely recognized for its role in determining muscle performance phenotypes. The R577X polymorphism results in the absence of α-actinin-3 protein in individuals homozygous for the X allele, leading to a shift toward a more oxidative muscle profile. This genotype has been associated with enhanced endurance capacity but reduced power output (Eynon et al., 2012; Hassan and Shady, 2020; Orysiak et al., 2015; Shang et al., 2010), although findings across studies remain inconsistent, highlighting the complexity of genotype-phenotype relationships (Bulgay et al., 2023b; Del Coso et al., 2024; Jacob et al., 2022; Papadimitriou et al., 2018). Another relevant gene is *CKM*, which encodes muscle-specific creatine kinase, a key enzyme in rapid ATP regeneration. The rs8111989 polymorphism has been associated with aerobic performance, with the A allele linked to improved oxidative capacity and higher VO_2_max (Zhou et al., 2006; Fedotovskaia et al., 2012). These findings have been supported by meta-analytical evidence, reinforcing the role of energy buffering systems in endurance physiology (Chen et al., 2017). Together, these genes illustrate how genetic variation can influence the balance between oxidative and glycolytic muscle function, ultimately shaping athletic specialization.

#### 3.2.2 Genetic regulation of muscle structure and adaptive remodeling

Beyond contractile function, genetic factors also influence muscle structural integrity and its capacity to adapt to training stimuli. Recent genome-wide association studies have expanded the spectrum of candidate genes involved in endurance performance, identifying novel variants linked to both muscle function and systemic physiological traits (Al-Khelaifi et al., 2020). The *MYBPC3* gene, traditionally associated with cardiac muscle structure, has been linked to higher VO_2_max values in elite athletes through its rs1052373 polymorphism, with the GG genotype showing a significant association with increased VO_2_max in elite athletes (Al-Khelaifi et al., 2020). This finding suggests a potential cross-talk between cardiac and skeletal muscle adaptations in endurance contexts. Similarly, The rs7564856 polymorphism near the *SPEG-β* gene has been associated with oxidative muscle fiber composition and endurance athlete status. The G allele correlates with increased SPEG-β expression in the rat gastrocnemius and a higher proportion of type I fibers in the human vastus lateralis, reinforcing its potential role in promoting oxidative muscle phenotypes (Kusić et al., 2020). This variant has also been associated with improved pulmonary function indices, further supporting the integrative nature of endurance-related traits (Kichaev et al., 2019). Structural proteins such as those encoded by the *COL5A1* gene also contribute to performance by influencing connective tissue properties. The rs12722 polymorphism has been associated with both flexibility and performance in ultra-endurance events, with the TT genotype linked to superior outcomes in ultra-endurance events (Posthumus et al., 2011; Brown et al., 2011). Overall, genetic variants affecting muscle structure and remodeling emphasize that endurance performance is not solely dependent on metabolic efficiency, but also on the capacity of tissues to withstand and adapt to repeated physiological strain.

### 3.3 Genetic determinants of metabolic regulation and energy efficiency

Energy metabolism lies at the core of endurance performance, governing the capacity to produce, utilize, and sustain ATP generation over extended periods. This process involves a tightly regulated network of metabolic pathways coordinating carbohydrate, lipid, and protein utilization in response to exercise demands (Santos-Concejero et al., 2014; Ama et al., 1985).

During prolonged effort, metabolic flexibility, the ability to switch efficiently between energy substrates, becomes a key determinant of performance. Enhanced lipid oxidation, glycogen sparing, efficient lactate clearance, and hormonal regulation collectively contribute to delaying fatigue and optimizing energy availability. These processes are, in part, genetically determined, with multiple variants influencing metabolic pathways, mitochondrial function, and substrate transport (Lucia et al., 2005; Wallace, 2010; Kikuchi et al., 2017).

#### 3.3.1 Genetic regulation of energy metabolism pathways

Genes involved in transcriptional regulation of metabolism play a central role in endurance adaptation. Among them, the PPAR family and their coactivators represent key regulators of lipid metabolism and mitochondrial biogenesis. The *PPARA* gene, particularly the rs4253778 polymorphism, has been associated with elite endurance athlete status and superior performance in prolonged aerobic events, with the GG genotype linked to enhanced fatty acid oxidation and improved performance in long-duration activities (Ahmetov et al., 2006; Cocci et al., 2019; Ginevičienė et al., 2010; Maciejewska et al., 2011; Vegh et al., 2022). This aligns with the metabolic demands of endurance exercise, during which lipid utilization becomes increasingly important over prolonged exercise durations, contributing to energy supply and performance maintenance when glycogen availability declines. The *PPARGC1A* gene, encoding PGC-1α, is a master regulator of mitochondrial biogenesis and oxidative metabolism. The Gly482Ser polymorphism has been extensively studied, with the Gly allele generally associated with elite endurance athlete status, higher aerobic capacity (VO_2_max), and superior performance in endurance-oriented disciplines (Jin et al., 2016; Eynon et al., 2010b; Gineviciene et al., 2014; Jacob et al., 2021), although conflicting findings suggest that its effects may vary depending on population and sport discipline (He et al., 2015; Hall et al., 2023; Bulğay et al., 2022; Valipour et al., 2022). Similarly, the rs7732671 G > C polymorphism of the *PPARGC1B* gene contributes to aerobic capacity through its role in energy metabolism (Ahmetov et al., 2009a), the 203Pro allele correlates with improved aerobic capacity, as reflected by oxygen pulse and energy capacity measurements (Akhmetov et al., 2009).

In addition to these transcriptional regulators, genes involved in energy metabolism pathways also contribute to endurance phenotypes. Notably, the *AMPD1* gene, which encodes adenosine monophosphate deaminase and plays a key role in energy turnover during exercise, has been associated with endurance athlete status (Kartibou et al., 2025). A study conducted in professional cyclists and elite endurance runners reported a higher frequency of the CC genotype compared with controls, suggesting a potential advantage in sustained energy metabolism (Varillas Delgado et al., 2020). Additional genes involved in metabolic efficiency include *UCP2* and *UCP3*, which regulate mitochondrial coupling and energy expenditure. Endurance-associated alleles of these genes have been shown to increase in frequency with higher athletic status, suggesting a role in optimizing energy efficiency (Ahmetov et al., 2008). Conversely, the *FTO* gene illustrates the complexity of interpreting genetic associations in sports genomics. Although *FTO* is one of the strongest obesity-associated loci identified in human genome-wide association studies, with associations with adiposity and body composition reported predominantly in populations of European ancestry, it has also been implicated in energy balance regulation. Some studies have reported associations between *FTO* variants and athletic traits (Zmijewski and Leońska-Duniec, 2021; Heffernan et al., 2017; Guilherme et al., 2019). These associations may reflect indirect effects mediated by differences in body composition, metabolic characteristics, or sport-specific selection processes rather than a direct modulation of endurance physiology. Therefore, *FTO*-related findings should be interpreted within the broader context of the polygenic architecture of endurance performance and considered as potential contributors rather than independent determinants. This variability reinforces the importance of population-specific analyses and integrative approaches when interpreting genetic data (Eynon et al., 2013a). Notably, recent GWAS findings, such as the identification of the *GALNTL6* rs558129 polymorphism, provide further evidence for the polygenic nature of endurance performance, with modest but significant contributions to athlete status (Rankinen et al., 2016).

#### 3.3.2 Genetic influences on mitochondrial function

Mitochondrial function is a fundamental determinant of endurance performance, providing the cellular energy required for sustained aerobic exercise. The nuclear-encoded *TFAM* gene plays a critical role in mitochondrial DNA maintenance and transcription (Theilen et al., 2017). The *TFAM* rs1937 polymorphism has been associated with higher aerobic capacity and a greater proportion of oxidative (type I) muscle fibers, with the Thr allele reported more frequently among individuals exhibiting these phenotypes. However, as muscle fiber composition is modifiable by training, this association may reflect a genetic contribution to inter-individual differences in fiber composition rather than a fixed determinant of fiber type (Ahmetov et al., 2009a; Akhmetov et al., 2010).

Both nuclear and mitochondrial genomes contribute to the regulation of mitochondrial function and energy metabolism. Mitochondrial DNA haplogroups represent distinct maternal lineages defined by characteristic combinations of inherited mitochondrial variants. Their potential relevance to endurance performance may arise from differences in mitochondrial bioenergetics, particularly oxidative phosphorylation, ATP production, and reactive oxygen species generation (Maruszak et al., 2014). Mitochondrial haplogroups have been associated with endurance performance, with haplogroup H, particularly H11, reported more frequently among elite endurance athletes, potentially reflecting differences in mitochondrial bioenergetics and ATP production efficiency (Maruszak et al., 2014). In contrast, haplogroup K has been associated with reduced oxidative capacity, highlighting the potential functional relevance of mitochondrial genetic variation (Wallace, 2010; Niemi and Majamaa, 2005).

Associations between mitochondrial haplogroups and endurance-related phenotypes require cautious interpretation. Mitochondrial haplogroups exhibit strong geographic structuring and reflect population history, migration patterns, and founder effects. Consequently, observed associations between specific haplogroups and endurance athlete status may partly reflect ancestry-related population structure rather than direct functional effects on performance. Moreover, functional evidence supporting causal links between most mitochondrial haplogroups and endurance adaptation remains limited. Future investigations should combine mitochondrial genomics with ancestry-adjusted analyses and functional studies to better distinguish true biological effects from demographic confounding factors. Furthermore, the transferability of mitochondrial genetic associations across ethnically diverse populations remains insufficiently explored and should be evaluated in large multi-ancestry cohorts.

#### 3.3.3 Genetic regulation of metabolite transport and fatigue resistance

Efficient transport of metabolic by-products is essential for maintaining performance during prolonged exercise. The *MCT1* gene, which encodes a key lactate transporter, plays a central role in this process. The Glu490Asp polymorphism, particularly the Glu allele, has been associated with higher VO_2_max values and enhanced lactate clearance capacity, both of which are important determinants of prolonged aerobic exercise performance (Guilherme et al., 2021; Santos-Concejero et al., 2014; Ama et al., 1985; Kohn et al., 2007). By facilitating the removal and reutilization of lactate, this variant contributes to delaying metabolic acidosis and sustaining muscular activity (Santos-Concejero et al., 2014). While carriers of the other mutated allele show higher post-exercise blood lactate concentrations, which may limit their performance (Kikuchi et al., 2017; Cupeiro et al., 2010; Cupeiro et al., 2012; Fedotovskaya et al., 2014).

### 3.4 Genetic determinants of immune and inflammatory responses in endurance athletes

The capacity to tolerate high training loads and recover efficiently is a defining feature of elite endurance athletes, relying heavily on the precise regulation of immune and inflammatory processes. Exercise-induced muscle damage triggers a transient inflammatory response that is essential for tissue repair and adaptation (Baskerville et al., 2024). However, this response must remain tightly controlled, as excessive or prolonged inflammation can impair recovery, increase susceptibility to illness, and contribute to overtraining syndromes (Baskerville et al., 2024). From a biological perspective, endurance performance depends on the ability to maintain a delicate balance between pro-inflammatory and anti-inflammatory signaling, while simultaneously managing oxidative stress generated during prolonged exercise (Baskerville et al., 2024). Genetic variability in immune regulation pathways plays a key role in shaping this balance, influencing both recovery kinetics and long-term adaptation (Ahmetov et al., 2009a; Eynon et al., 2010a).

#### 3.4.1 Genetic influences on immune modulation

Genes involved in immune signaling and transcriptional regulation contribute to inter-individual variability in endurance performance. The *NFATC4* gene, a key regulator of immune and muscle-related gene expression, has been associated with endurance athlete status, with the Gly160 allele linked to enhanced aerobic capacity (Ahmetov et al., 2009a). Notably, the frequency of this allele varies considerably across populations, suggesting potential ethnic-specific effects (Ahmetov et al., 2009a). Similarly, the *PRDM1* gene, which plays a role in immune cell differentiation and transcriptional repression, has been implicated in endurance performance (Hall et al., 2021a). The rs10499043 polymorphism has been associated with improved marathon performance, with carriers of the T allele exhibiting faster race times compared to individuals with the CC genotype (Hall et al., 2021a). In addition, the *IL6* gene, which encodes the exercise-responsive cytokine interleukin-6, has been implicated in endurance performance and may contribute to training adaptation through its role in regulating exercise-induced inflammatory responses, energy metabolism, and substrate utilization. The rs1800795 (-174G/C) polymorphism has attracted particular attention, with studies reporting a higher prevalence of the C allele and CC genotype among elite long-distance swimmers compared with runners and non-athletic controls (Ben-Zaken et al., 2017). Furthermore, the GC genotype and C allele have been associated with superior performance in male cross-country skiers (Kazanci et al., 2023). These findings suggest that *IL6* genetic variation may influence sport-specific endurance phenotypes, particularly long-distance swimming performance and cross-country skiing performance, through modulation of inflammatory responses and exercise adaptation.

#### 3.4.2 Genetic regulation of oxidative stress and inflammation

Oxidative stress is an inevitable consequence of sustained aerobic metabolism, and the ability to counterbalance reactive oxygen species production is critical for maintaining cellular integrity and performance (Eynon et al., 2010a; İpekoğlu et al., 2024). Genetic variants involved in antioxidant defense systems therefore represent important determinants of endurance capacity. The *NRF2* gene, a master regulator of antioxidant responses, has been associated with endurance performance in several studies. The rs12594956 polymorphism, particularly the AA genotype, has been reported at higher frequencies in endurance athletes compared to sprinters and non-athletes (Eynon et al., 2010a). This genotype is thought to enhance the transcription of antioxidant enzymes, thereby improving mitochondrial efficiency and supporting higher VO_2_max values (Eynon et al., 2013b). Importantly, these findings have been consistently replicated across multiple cohorts and reinforced by recent meta-analytical evidence (İpekoğlu et al., 2024; Eynon et al., 2013b).

### 3.5 Genetic determinants of neurological function and stress responsiveness

Endurance performance is not solely a physiological phenomenon but also depends on the integration of neural control and psychological resilience. The nervous system plays a central role in coordinating motor output, regulating fatigue perception, and adapting to the cognitive and emotional demands of competition (Tossici et al., 2024; Geisler et al., 2021). Genetic factors influencing neural signaling, synaptic plasticity, and hormonal stress responses contribute to inter-individual differences in performance, particularly under conditions of prolonged physical and psychological stress (Ahmetov et al., 2015; Bulgay et al., 2023a).

#### 3.5.1 Genetic influences on neurological regulation

Genomic studies have identified novel genes involved in neuromuscular coordination and endurance performance. The *RBFOX1* gene, which regulates alternative RNA splicing in muscle, cardiac, and neuronal tissues, has been associated with endurance athlete status. The rs7191721 polymorphism, particularly the G allele, is linked to improved aerobic capacity and muscle function, highlighting the importance of post-transcriptional regulation in performance phenotypes (Ahmetov et al., 2015). Additionally, variants in the *TRPM2* gene, involved in calcium signaling and cellular stress responses, have been associated with endurance traits. The *TRPM2* rs1785440 polymorphism has been associated with endurance athlete status and higher VO_2_max, with the G allele being more frequent among endurance athletes. This association may reflect the role of TRPM2 in Ca^2+^ dependent signaling and its potential regulation of mitochondrial function and muscle fiber-type adaptations. Such adaptations may enhance oxidative capacity and fatigue resistance, thereby supporting endurance performance (Bulgay et al., 2023a).

#### 3.5.2 Genetic regulation of stress and hormonal responses

Adaptation to physical and psychological stress is a critical determinant of endurance performance. Genes involved in adrenergic signaling pathways play a key role in modulating cardiovascular, metabolic, and hormonal responses to exercise. The *ADRB2* gene, particularly the Arg16Gly (rs1042713) polymorphism, has been associated with endurance athlete status, with the Arg16 allele more frequently observed in endurance athletes (Wolfarth et al., 2007; Tsianos et al., 2010). Similarly, the *ADRB3* gene has been linked to elite endurance athlete status through its role in energy metabolism and lipolysis. The rs4994 polymorphism has shown significant associations with elite endurance status across different populations, reinforcing its contribution to metabolic efficiency (Santiago et al., 2011; Yang et al., 2021). In addition, the rs7144481 polymorphism of the *TSHR* gene, and more specifically the C allele, was linked to increased aerobic capacity and is frequently found in endurance athletes, indicating a role in muscle metabolism and angiogenesis (Ahmetov et al., 2015).

### 3.6 Genetic determinants of cellular function and longevity

At the cellular level, endurance performance depends on the ability to maintain genomic stability, preserve mitochondrial efficiency, regulate oxidative stress, and sustain long-term physiological adaptation. These mechanisms are particularly relevant in endurance athletes, who are repeatedly exposed to metabolic stress, inflammation, and exercise-induced cellular damage. Importantly, the relationship between endurance performance and longevity should be interpreted cautiously, as endurance-associated genetic variants are not necessarily direct longevity determinants. Rather, several molecular pathways involved in cellular maintenance, stress resistance, and metabolic regulation may contribute to both endurance adaptation and healthy aging through partially overlapping mechanisms (Semenova et al., 2022; Carapeto and Aguayo-Mazzucato, 2021).

#### 3.6.1 Genetic regulation of cell cycle and genomic stability

Genes involved in cell cycle control and DNA integrity contribute to muscle adaptation and performance. The *CDKN1A* gene, through the rs236448 polymorphism, has been associated with muscle fiber composition, with the A allele being associated with a higher proportion of oxidative (type I) muscle fibers and endurance-oriented athlete status, whereas the C allele has been associated with a greater proportion of fast-twitch fibers and strength-related traits (Semenova et al., 2022). Similarly, the NUP210 gene, involved in nuclear-cytoskeletal interactions and muscle regeneration, has been associated with improved muscular endurance, further supporting the role of cellular architecture in performance (Bulgay et al., 2023a). These findings suggest that variants involved in cellular integrity and muscle remodeling may influence endurance capacity by promoting efficient adaptation to repeated physiological stress. However, whether these mechanisms directly contribute to longevity remains unclear and requires further investigation.

#### 3.6.2 Genetic influences on cellular repair and longevity pathways

Longevity-related pathways may indirectly influence endurance performance by regulating mitochondrial function, energy metabolism, oxidative stress resistance, and cellular repair mechanisms. The *SIRT1* gene, a key regulator of cellular metabolism and aging-related processes, has been associated with higher VO_2_max values and a greater proportion of oxidative muscle fibers through its interaction with PGC-1α signaling pathways (Bulgay et al., 2023a). Variants such as rs41299232 have been linked to increased VO_2_max and a higher proportion of oxidative muscle fibers, suggesting a role in enhancing mitochondrial function and adaptive capacity (Bulgay et al., 2023a). Nevertheless, the association between *SIRT1*-related genetic variation and endurance performance should not be interpreted as evidence that these variants directly promote longevity in athletes. Instead, they may reflect shared biological mechanisms underlying both exercise adaptation and cellular resilience. Moreover, potential trade-offs may exist between mechanisms favoring high-performance adaptation, energetic investment, tissue remodeling, and long-term maintenance.

#### 3.6.3 Integrated genetic regulation of cellular function

The rs7181866 polymorphism of the GABPB1 gene, located in intron 3, has been linked to sports performance, particularly maximal oxygen consumption (VO_2_max) in response to endurance training. Although intronic, rs7181866 may influence *GABPB1* function through regulatory effects on gene transcription and expression. He et al. (2007) showed that the ATG haplotype, defined by the combined presence of three SNPs (rs12594956, rs8031031, and rs7181866), was associated with higher VO_2_max values in previously untrained Chinese Han men undergoing an 18-week endurance training program. Moreover, carriers of the ATG haplotype exhibited a 57.5% greater training response in VO_2_ max compared with non-carriers (p = 0.006) (He et al., 2007). Furthermore, Eynon et al. (2009) found that the AG genotype of rs7181866 was associated with significantly higher GABPB1 mRNA expression in peripheral blood mononuclear cells (PBMCs) and was more prevalent among Israeli elite endurance athletes, suggesting an association with endurance athlete status rather than with a specific physiological performance trait, particularly in elite cohorts. They suggested that this genotype, together with the Gly/Gly + Gly/Ser genotypes of *PPARGC1A* gene, could be the “optimal genotype” for endurance athletes (Eynon et al., 2009). This association was confirmed by Maciejewska-Karłowska et al. (2012), who observed an overrepresentation of the AG genotype and G allele in Polish endurance athletes, particularly rowers, compared with sedentary controls (Maciejewska-Karłowska et al., 2012). In contrast, the AA genotype was present in all rugby players, suggesting that it may be predominant in this type of athlete, but not the sole determinant of endurance performance. These results underline the complexity of genetic and environmental interactions influencing sports performance (Yilmaz et al., 2023). Moreover, Ahmetov’s 2015 study was the first to identify an association between *NFIA-AS2* gene polymorphism and aerobic performance, as well as elite endurance athlete status, using a two-stage genome-wide association approach (Ahmetov et al., 2015). Specifically, SNP rs1572312 (allele C) of the *NFIA-AS2* gene was associated with high aerobic capacity and enhanced erythropoiesis, potentially affecting hemoglobin, reticulocyte and erythrocyte levels via *NFIA* gene expression (Ahmetov et al., 2015). Similarly, the 5I polymorphism of the *PPP3R1* promoter was associated with improved aerobic capacity and maximum energy production (Ahmetov et al., 2009a).

### 3.7 Genetic determinants of water balance and ion transport

Maintaining fluid and electrolyte balance is essential for sustaining endurance performance, particularly under conditions of prolonged exercise and thermal stress (de la Igles et al., 2020). The *AQP1* gene, encoding a key water channel protein, has been associated with endurance performance through the rs1049305 polymorphism. The C allele has been linked to superior performance in long-distance events, potentially due to enhanced cellular water transport and improved resistance to dehydration (Rivera and Fahey, 2019). These findings have been reported across different athletic populations and are further supported by experimental evidence from AQP1-knockout mice, which exhibited markedly reduced voluntary exercise tolerance compared with wild-type mice under normoxic, hypoxic, and hyperoxic conditions (Rivera et al., 2020; de la Igles et al., 2020; Saunders et al., 2015; Rivera et al., 2011; Xu et al., 2010).

### 3.8 Genetic determinants of iron metabolism and oxygen transport

Iron metabolism is an essential component of endurance performance, as iron contributes to hemoglobin and myoglobin synthesis, which are required for efficient oxygen transport and utilization during prolonged exercise (Thakkar et al., 2021). Adequate iron availability may support aerobic capacity by improving tissue oxygenation and reducing exercise-induced fatigue (Semenova et al., 2020; Thakkar et al., 2021). Therefore, genetic variants involved in iron regulation may contribute to inter-individual differences in endurance-related traits. Among these, the *HFE* gene has received attention due to its role in iron homeostasis, with the rs1799945 (H63D) polymorphism being associated with endurance-related phenotypes in several populations (Deugnier et al., 2002). Semenova et al. reported that CG/GG genotypes of H63D were more frequent among Russian and Japanese endurance athletes compared with controls, suggesting a possible association between *HFE* variation and endurance status (Semenova et al., 2020). Similarly, studies in French athletes reported associations between the G allele and hematological parameters, including hematocrit and hemoglobin concentration, which are relevant to aerobic performance (Hermine et al., 2015). Furthermore, genome-wide analyses have provided additional evidence supporting the involvement of *HFE*-related variation in endurance-associated traits (Astl et al., 2016). However, these findings should be interpreted cautiously, as *HFE* rs1799945 represents one of many genetic contributors within the polygenic architecture of endurance performance, and its effect is likely influenced by environmental factors, training status, and interactions with other metabolic pathways.

## 4 Genetic determinants of inter-individual variability in response to aerobic training

Inter-individual variability in response to aerobic training represents a major challenge in exercise physiology, as individuals exposed to similar training stimuli may exhibit different magnitudes of physiological adaptation. While some athletes demonstrate substantial improvements in aerobic capacity, others may show more modest changes, highlighting the limitations of uniform training approaches (Fyfe et al., 2014). The magnitude of these adaptations may also vary according to the specific training modality, intensity, and duration. Genetic factors may contribute to this variability by influencing multiple physiological pathways, including body composition, insulin sensitivity, cardiovascular responses, and particularly training-induced changes in VO_2_max. Importantly, this suggests that genetic background contributes not only to baseline performance but also to the capacity for physiological adaptation (Bae et al., 2007; Duoqi et al., 2015; Weiss et al., 2005; Jayewardene et al., 2016). Recent evidence has also emphasized the role of genetic variants involved in oxidative stress regulation. Polymorphisms in detoxification-related genes such as *GSTM1*, *GSTP* (rs1695), and *CYP2D6* (rs3892097) have been associated with improved recovery capacity and are more frequently observed in elite athletes (Table 2). However, findings remain inconsistent. For instance, while some studies found no significant association between GSTP variants and specific measures of exercise performance, others reported that carriers of the G allele (AG + GG) showed greater improvements in VO_2_max and maximal ventilation, as well as greater increases in lean mass, compared with AA homozygotes (Varillas Delgado et al., 2019; Zarebska et al., 2014).

**TABLE 2:** Genetic markers associated with variability in individual response to aerobic training.

Gene	Gene name	Key SNPs	Locus	Related allele, (References)
*GSTP*	Glutathione S-transferase Pi	11q13.2	rs1695A > G	G, (Zarebska et al., 2014)
*CYP2D6*	Cytochrome P450 family 2 subfamily D member 6	22q13.2	rs3892097 G>A	G, (Varillas Delgado et al., 2019)
*GSTM1*	Glutathione S-transferase mu	1p13.3	Functional(+)/null(−)	null (−), (Varillas Delgado et al., 2019)

These discrepancies highlight the context-dependent nature of genetic effects and the importance of gene-environment interactions, including nutritional factors that may modulate oxidative stress and recovery (Close et al., 2016). Overall, current evidence remains limited by the focus on isolated polymorphisms, underscoring the need for integrative approaches combining genomics and other “omics” to better understand the biological basis of individual variability in training responses. Although genetic markers may eventually contribute to a more comprehensive understanding of inter-individual differences in adaptation to training, current evidence does not support their use for athlete selection or for prescribing training strategies based solely on genetic profiles.

## 5 Polygenic scores and cumulative genetic models of endurance performance

Although numerous genetic variants have been associated with endurance-related phenotypes, the effect size of most individual polymorphisms is generally small and insufficient to explain the remarkable physiological characteristics observed in elite endurance athletes. Endurance performance is increasingly recognized as a highly complex polygenic trait resulting from the cumulative contribution of numerous loci distributed across multiple biological pathways, including cardiovascular function, oxygen transport, mitochondrial biogenesis, substrate utilization, angiogenesis, inflammation, neuromuscular regulation, and recovery processes (Bıçakçı et al., 2024; Kim et al., 2022). Consequently, the traditional candidate-gene paradigm, which focuses on isolated polymorphisms, provides only a partial representation of the genetic architecture underlying endurance performance (Bıçakçı et al., 2024).

Recognition of this complexity has led to the development of cumulative genetic models designed to capture the combined effects of multiple endurance-associated alleles. The original Total Genotype Score (TGS) model proposed by Williams and Folland incorporated 23 genetic polymorphisms previously associated with endurance-related phenotypes and was used to model the distribution of theoretical polygenic endurance profiles in a hypothetical population of one million individuals based on population genotype frequencies (Williams and Folland, 2008).

Subsequent studies adapted this approach using smaller, study-specific panels of variants. For example, Ruiz et al. evaluated a seven-polymorphism TGS comprising *ACE* I/D, *ACTN3* R577X, *AMPD1* Gln12Ter, *CKMM* 1170 bp/985 + 185 bp, *HFE* H63D, *GDF8* Lys153Arg, and *PPARGC1A* Gly482Ser in 46 world-class endurance athletes and 123 controls, all of Spanish Caucasian origin (Ruiz et al., 2009). The same seven-variant profile was subsequently evaluated in 196 elite Ironman triathletes from the 2008 Kona Ironman Championship, representing multiple continents (Grealy et al., 2015). In contrast, Neto et al. investigated a three-variant model comprising *ACTN3* R577X, *ACE* I/D, and *BDKRB2* −9/+9 in Brazilian long-distance swimmers (Neto et al., 2022). These findings indicate that cumulative genotype models may capture aspects of the polygenic architecture of endurance-related traits; however, the number and identity of variants included vary substantially between studies, and the predictive value of these scores has not been consistently demonstrated across athletic populations.

Accordingly, proposed “optimal genotype profiles” should be considered study-specific models rather than evidence for a broadly shared genetic profile of endurance performance. Indeed, even studies using the same seven-variant profile have reported heterogeneous relationships with athlete status or competitive performance. These observations further support the multifactorial nature of endurance performance and the importance of considering environmental, training, and population-specific factors alongside genetic variation (Ruiz et al., 2009; Grealy et al., 2015).

More recently, advances in GWAS have facilitated the transition from candidate-gene-based TGS models toward genome-wide polygenic scores (PGS) (Choi et al., 2020). Unlike traditional approaches that rely on a limited number of predefined variants, polygenic scores integrate information from hundreds, thousands, or even millions of genetic markers across the genome. This strategy better reflects the biological reality that complex human traits are influenced by a large number of variants, each contributing only a modest proportion of the overall phenotype (Choi et al., 2020). In the context of endurance performance, polygenic models have demonstrated superior predictive capacity compared with single-marker analyses, while also providing insights into the distributed genetic architecture of aerobic fitness, training responsiveness, and athlete status. Nevertheless, several limitations should be acknowledged. Most TGS and polygenic models have been developed using relatively small cohorts of elite athletes, raising concerns regarding statistical power, reproducibility, and cross-population transferability. Moreover, the predictive value of these models remains limited because endurance performance is strongly influenced by environmental factors, including training exposure, nutrition, recovery strategies, and other non-genetic determinants. Consequently, polygenic scores should be viewed as indicators of biological predisposition rather than deterministic predictors of athletic success.

## 6 Epigenetic regulation and endurance athlete status

Epigenetic regulation represents a critical interface between genetic predisposition and environmental exposure, enabling dynamic modulation of gene expression in response to stimuli such as exercise and physiological stress (Plaza-Diaz et al., 2022). Key mechanisms, including DNA methylation, histone modifications, and non-coding RNAs, play a central role in orchestrating transcriptional responses underlying endurance adaptation (Williams et al., 2025). In the context of endurance performance, these processes regulate fundamental pathways such as mitochondrial biogenesis, angiogenesis, muscle fiber plasticity, and post-exercise recovery. Unlike static genetic variants, epigenetic modifications are reversible and highly responsive to training load, providing a mechanistic basis for the rapid and sustained adaptations observed in athletes (Mallett, 2025; Barrès et al., 2012; Wardle et al., 2015; Baggish et al., 2011). Importantly, epigenetic plasticity may contribute to inter-individual variability in training responsiveness among athletes with similar genetic backgrounds. This highlights the relevance of integrating epigenetic insights into sports science to better understand the molecular mechanisms underlying adaptation and differences in training responses.

### 6.1 Exercise-induced DNA methylation

DNA methylation is one of the most extensively studied epigenetic mechanisms in skeletal muscle, acting primarily as a regulator of gene expression through transcriptional repression when located in promoter regions (Mallett, 2025). In the context of exercise, however, this mechanism exhibits dynamic and reversible behavior that may contribute to physiological adaptation. Early evidence from Barres et al. suggested that a single bout of acute exercise could induce rapid, gene-specific hypomethylation in skeletal muscle, including at metabolic genes such as *PPARGC1A*, *TFAM*, and *NRF1*. However, subsequent studies have yielded variable findings, suggesting that acute exercise-induced DNA methylation changes may be modest, gene-specific, and dependent on the timing of tissue sampling. Moreover, the original findings were obtained in previously sedentary adults rather than trained or elite athletes, and therefore cannot be directly extrapolated to athletic populations. Longer-term training studies provide stronger evidence for exercise-associated DNA methylation remodeling, supporting a potential role for epigenetic regulation in endurance adaptation (Barrès et al., 2012). Beyond metabolic genes, emerging data suggest that DNA methylation also interacts with classical genetic variants to influence performance-related phenotypes. Notably, methylation of the *ACE* gene promoter in skeletal muscle has been proposed as an epigenetic marker of endurance adaptation, capable of modulating gene expression and interacting with the I/D polymorphism to influence oxidative muscle phenotype (Martin et al., 2026).

#### 6.1.1 Epigenetic memory, long-term methylation signatures and gene-environment interactions

Beyond the acute methylation changes induced by a single exercise bout, accumulating evidence indicates that endurance training can produce persistent epigenetic remodeling in skeletal muscle. Repeated exercise exposure generates long-term DNA methylation signatures affecting genes involved in mitochondrial biogenesis, oxidative metabolism, angiogenesis, and muscle remodeling (Seaborne et al., 2018). These modifications may persist even after periods of detraining, supporting the concept of “epigenetic memory” (Seaborne et al., 2018; Pilotto et al., 2025; Turner et al., 2019). Such molecular memory has been proposed as a mechanism explaining the accelerated retraining responses frequently observed in previously trained individuals (Seaborne et al., 2018; Pilotto et al., 2025).

Importantly, considerable inter-individual variability exists in exercise-induced methylation responses. Athletes exposed to similar training regimens often display markedly different epigenetic profiles, suggesting that methylation dynamics are influenced by age, sex, nutritional status, environmental exposures, recovery strategies, and genetic background. Consequently, DNA methylation represents a key interface through which environmental factors shape endurance adaptation (Geiger et al., 2024; Widmann et al., 2019; Voisin et al., 2015).

Increasing evidence also supports interactions between genetic and epigenetic variation. Genetic polymorphisms can influence local methylation patterns through methylation quantitative trait loci (meQTLs), thereby modulating gene expression responses to training stimuli (Gaunt et al., 2016). Such genotype-epigenotype interactions may contribute to the substantial variability observed in endurance-related phenotypes. Furthermore, population-specific methylation signatures associated with ancestry, environmental history, and lifestyle factors may partly explain inconsistencies across athlete-genetics studies and highlight the importance of integrating population epigenetics into future endurance research (Voisin et al., 2015).

### 6.2 Histone modifications and gene activation

Histone modifications represent a central epigenetic mechanism regulating gene expression through dynamic changes in chromatin structure (Plaza-Diaz et al., 2022). Post-translational modifications (PTMs) such as acetylation, methylation, and phosphorylation, collectively referred to as the “histone code”, modulate chromatin accessibility and recruit transcriptional machinery in response to environmental stimuli, including physical exercise (Plaza-Diaz et al., 2022). In skeletal muscle, exercise induces rapid and coordinated remodeling of histone marks that favor transcriptional activation (Plaza-Diaz et al., 2022). Endurance exercise has been associated with increased histone acetylation in skeletal muscle (e.g., H3K36ac), promoting chromatin relaxation and facilitating the expression of genes involved in oxidative metabolism and mitochondrial function (McGee et al., 2009). Similarly, phosphorylation of histone H3, mediated by signaling pathways such as AMPK and CaMK-II, occurs rapidly in skeletal muscle following exercise and contributes to early transcriptional activation (Yu et al., 2003).

Histone methylation further refines this regulatory landscape, with its effects depending on the specific residue and methylation state. Exercise-induced increases in activating marks, such as H3K4 methylation in promoter regions, have been linked to enhanced transcription of metabolic and muscle-specific genes (Plaza-Diaz et al., 2022). Notably, endurance training has been shown to drive epigenetic reprogramming toward a more oxidative phenotype, characterized by increased expression of PGC-1α, enhanced mitochondrial biogenesis, and a shift from fast- to slow-twitch muscle fibers, partly mediated through ROS/AMPK-dependent activation of histone-modifying enzymes (Li et al., 2024). Collectively, these modifications establish a chromatin environment that supports sustained gene activation and muscle adaptation, highlighting the pivotal role of histone dynamics in translating exercise stimuli into functional genomic responses (Plaza-Diaz et al., 2022; Li et al., 2024).

#### 6.2.1 Chromatin remodeling and transcriptional plasticity

In addition to DNA methylation and histone modifications, exercise-induced chromatin remodeling has emerged as a major regulator of endurance adaptation (de Smalen et al., 2025). Chromatin remodeling complexes dynamically alter nucleosome positioning and chromatin accessibility, facilitating the recruitment of transcription factors involved in oxidative metabolism, mitochondrial biogenesis, angiogenesis, and muscle fiber specification (McGee and Walder, 2017; Lindholm et al., 2014). Endurance training promotes the establishment of a more permissive chromatin landscape, enabling sustained activation of exercise-responsive genes (McGee and Walder, 2017; Lindholm et al., 2014).

Studies suggest that repeated exercise exposure induces coordinated modifications across multiple layers of chromatin regulation, including changes in chromatin accessibility, enhancer activity, and three-dimensional genome organization (Barrès et al., 2012; Zheng et al., 2025). These alterations may contribute to transcriptional plasticity and influence subsequent responses to exercise stimuli (Voisin et al., 2015; Zheng et al., 2025). However, much of the current mechanistic evidence for exercise-induced chromatin remodeling comes from experimental studies in non-athletic or recreationally active populations and should therefore not be directly extrapolated to elite endurance athletes. Although differences in baseline epigenetic profiles have been reported between trained and untrained individuals, the extent to which training-induced epigenetic remodeling occurs specifically in athletes, and how it contributes to inter-individual variability in adaptation, remains insufficiently characterized. Therefore, chromatin remodeling represents a promising mechanism linking training stimuli to molecular adaptation, but athlete-specific longitudinal studies are needed to establish its role in endurance performance and training adaptation.

### 6.3 Role of microRNAs in endurance adaptation

MicroRNAs (miRNAs) are small non-coding RNAs, typically ∼18–24 nucleotides in length, that regulate gene expression through post-transcriptional mechanisms, primarily by guiding the RNA-induced silencing complex (RISC) to complementary sequences within target mRNAs. Although binding to the 3′untranslated region (3′UTR) is the most commonly described mechanism, miRNAs can also interact with other regions of target transcripts and influence mRNA stability and translation. In skeletal muscle, they play a pivotal role in coordinating key biological processes, including myogenesis, regeneration, metabolic regulation, and fiber-type specification (Clauss et al., 2016; Baggish et al., 2014). Endurance exercise induces specific and reproducible changes in miRNA expression profiles, highlighting their potential involvement in exercise adaptation. Several miRNAs have been associated with pathways related to mitochondrial biogenesis, oxidative metabolism, angiogenesis, and muscle fiber specification. However, while some miRNAs have demonstrated mechanistic roles in these processes, for many exercise-responsive miRNAs, current evidence primarily supports their potential as molecular indicators of training-related physiological changes rather than as validated biomarkers for individual training status or performance. Their interpretation remains challenging because miRNA responses can vary according to training modality, exercise intensity, timing of sampling, tissue or biofluid analyzed, and inter-individual variability. Notably, emerging evidence highlights the role of circulating miRNAs, particularly those encapsulated in extracellular vesicles, as mediators of inter-tissue communication, contributing to systemic metabolic adaptation (Clauss et al., 2016; Baggish et al., 2014; Fernandez-Sanjurjo et al., 2024).

Recent studies have identified distinct miRNA signatures associated with training status. For instance, reduced levels of miR-16-5p, miR-19a-3p, and miR-451a have been reported in trained athletes, while miR-25-3p appears specifically downregulated in endurance athletes, suggesting a role in chronic exercise adaptation (Fernandez-Sanjurjo et al., 2024). Conversely, acute endurance challenges, such as ultramarathon events, induce transient increases in miRNAs including miR-125a-5p, miR-126, and miR-223, alongside decreases in miR-15b, reflecting dynamic regulation in response to physiological stress (Marszalek et al., 2021).

Collectively, these findings underscore the growing importance of miRNAs in exercise physiology. However, a distinction should be made between miRNAs serving as biomarkers of exercise exposure and those directly mediating adaptive responses. While several exercise-responsive miRNAs have been mechanistically linked to angiogenesis, mitochondrial function, muscle remodeling, and fiber-type transitions, evidence for a causal role remains limited for many candidates. Therefore, further functional and longitudinal studies are required to clarify their contribution to endurance adaptation and training responsiveness (Table 3).

**TABLE 3:** Characteristics of microRNAs modulated by endurance exercise in athletes.

Studied population (references)	Sample type	Methodology	miRNA	Regulation	Potential function
Ultramarathon runners (Eyileten et al., 2022)	Plasma	qRT-PCR	miR-125a-5p	↑ post-race (p = 0.007); negatively correlated with maximum lactate	Regulation of angiogenesis, inflammation, and energy metabolism
			miR-126	↑ post-race (p = 0.001)	Endothelium, angiogenesis, vascular protection
			miR-223	↑ post-race (p = 0.014)	Inflammation, macrophage polarization, metabolic regulation
			miR-15b	↓ post-race (p = 0.028)	Glucose metabolism, insulin signaling
Half-marathon (Danese et al., 2018)	Plasma	RT-PCR	miR-206	↑ significantly post-run (1.36 vs. 0.63 × 10^−4^; p = 0.001)	Satellite cell activation, muscle regeneration, fiber type regulation
Endurance athletes, strength athletes and controls (Wardle et al., 2015)	Plasma	qRT-PCR	miR-222	↑ in END, ↓ in STR (p = 0.028)	Angiogenesis, vascular remodeling
			miR-221	↑ in END vs. STR (1.51-fold, p = 0.026)	Angiogenesis, endothelial regulation
Marathon runners vs. elite college rowers (Baggish et al., 2014)	Plasma	RT-PCR	miR-1	↑ at finish (∼22-fold), ↓ to baseline at 24 h	Muscle hypertrophy, contractile force regulation
			miR-133a	↑ at finish (∼18-fold), ↓ to baseline at 24 h	Myocyte proliferation/differentiation, muscle adaptation
			miR-499-5p	↑ modestly at finish (∼2.3-fold), variable at 24 h	Fiber type determination, endurance regulation
			miR-146	↑ at finish (∼3.3-fold), ↓ to baseline at 24 h	Inflammatory signaling, NF-κB pathway modulation
			miR-208	↑ strongly at finish (∼9-fold), ↓ to baseline at 24 h	Cardiac muscle stress response, hypertrophy regulation
Competitive rowers (Baggish et al., 2011)	Plasma	RT-PCR	miR-21	↑ after acute exercise before training, but not after training	Inflammation, cardiac/skeletal muscle contractility
			miR-221	↑ after acute exercise before training, but not after training	Angiogenesis, endothelial regulation

↑: upregulated; ↓: downregulated, END: endurance, STR: strength.

## 7 Current limitations and future directions in endurance genomics

### 7.1 Outstanding questions and future research priorities in endurance genomics

Despite substantial advances in sports genomics, several fundamental questions remain unresolved and continue to limit our understanding of the biological basis of elite endurance performance. First, the extent to which identified genetic variants contribute to elite endurance status across diverse populations remains unclear. Many candidate gene associations have shown inconsistent replication across studies, highlighting the need for larger and more ethnically diverse cohorts to validate existing findings and identify population-specific genetic architectures. Second, the relative contribution of genetic predisposition versus environmental influences remains poorly quantified. Endurance performance emerges from complex interactions among genetic factors, training load, nutrition, recovery strategies, and environmental exposures. Elucidating these gene-environment interactions represents a critical challenge for future research. Third, the molecular mechanisms linking genetic variation to physiological adaptation are not yet fully understood. While numerous variants have been associated with endurance-related traits, the biological pathways through which they influence mitochondrial function, oxygen transport, muscle remodeling, and recovery processes remain incompletely characterized. Fourth, the determinants of inter-individual variability in training responsiveness require further investigation. Considerable differences exist in the magnitude of adaptation achieved following identical training programs, yet the genetic and epigenetic factors underlying high- and low-responder phenotypes remain largely unresolved. Fifth, the integration of genomics with epigenomics, transcriptomics, proteomics, metabolomics, and microbiome data remains in its infancy. Future studies should move beyond the identification of isolated genetic associations and adopt integrative systems biology frameworks that consider endurance performance as an emergent phenotype resulting from the interaction of multiple biological layers. In this context, genetic variants may influence the baseline molecular capacity of key pathways involved in mitochondrial biogenesis, energy metabolism, oxygen utilization, inflammation, and muscle remodeling, whereas training exposure acts as a major environmental stimulus regulating epigenetic modifications and transcriptional responses. Understanding how genotype, epigenotype, transcriptome, and environmental factors interact over time will be essential to explain individual variability in endurance adaptation and performance outcomes. Finally, the translation of genomic discoveries into practical applications for athlete development remains an important challenge. The scientific validity, predictive accuracy, and ethical implications of genetic testing for talent identification and performance optimization require careful evaluation before widespread implementation in sport.

Addressing these questions will be essential for advancing endurance genomics from the identification of genetic associations toward a comprehensive understanding of the biological mechanisms underlying elite athletic performance.

### 7.2 Population structure, ancestry, and transferability of athlete-genetics associations

A major limitation of current sports genomics research is the insufficient consideration of population structure and ancestry-related genetic variation. Most athlete-genetics studies have been conducted in relatively small cohorts from geographically restricted populations, often of European or East Asian origin. Consequently, the transferability and reproducibility of reported genetic associations across populations remain uncertain.

The frequency of several endurance-associated variants differs substantially among continental populations. Variants including *ACE* rs1799752, *ACTN3* rs1815739, *HFE* rs1799945, *PPARA* rs4253778, and *PPARGC1A* rs8192678 exhibit marked allele-frequency heterogeneity across African, European, Asian, and admixed populations. Such differences may partly explain the inconsistent replication of athlete-genetics associations reported across studies. Importantly, failure to replicate a genetic association in a different population does not necessarily imply the absence of a biological effect, but may reflect differences in allele frequencies, linkage disequilibrium patterns, environmental exposures, or genetic background.

Population stratification represents another major challenge. If athlete and control groups differ in ancestry composition, spurious associations may arise due to systematic allele-frequency differences rather than true biological effects. Therefore, genetic associations should always be interpreted within a population-genetics framework.

This issue has been highlighted by Massidda et al.(2012), who investigated the distribution of *ACE* and *ACTN3* polymorphisms in elite Italian athletes (Massidda et al., 2012). Their findings emphasized that genotype frequencies must be interpreted relative to the genetic structure of the reference population, demonstrating that athlete-genetics associations cannot be evaluated independently of ancestry-related genetic background.

Future sports genomics studies should increasingly adopt methodologies routinely applied in population genetics, including ancestry inference, principal component analysis-based correction for population structure, admixture analysis, local ancestry mapping, and cross-ancestry validation of genetic predictors. These approaches are essential for distinguishing true biological associations from population-specific artifacts and for improving the reproducibility and transferability of genetic findings across diverse athletic populations.

## 8 Conclusion

The relationship between genetics and endurance performance represents a rapidly evolving area of research with important implications for sports science and performance optimization. This review identified more than 44 genetic polymorphisms associated with diverse endurance-related phenotypes, including elite endurance athlete status, VO_2_max, aerobic capacity, training responsiveness, oxidative muscle fiber composition, and sport-specific performance outcomes. Among these, the most consistently associated variants include the I allele of *ACE* (rs1799752), the G allele of *HFE* (rs1799945), the G allele of *PPARA* (rs4253778), and the Gly allele of *PPARGC1A* (rs8192678), alongside emerging markers such as CDKN1A rs236448 A, MYBPC3 rs1052373 G, *NFIA-AS2* rs1572312 C, and *GABPB1* rs12594956 A. Collectively, these variants highlight the polygenic nature of endurance performance and its dependence on interconnected physiological pathways.

However, genetic predisposition alone is insufficient to explain the substantial inter-individual variability observed in training responsiveness. Epigenetic mechanisms, including DNA methylation, histone modifications, and microRNAs (miR-222, miR-221, miR-206), play a pivotal role in regulating gene expression in response to exercise stimuli. These mechanisms dynamically modulate key biological pathways involved in muscle remodeling, mitochondrial biogenesis, and oxidative metabolism, particularly through signaling networks such as ROS/AMPK and PGC-1α. By influencing the functional expression of genetic variants, epigenetic regulation acts as a critical interface between genotype and environment, shaping endurance-related phenotypes beyond inherited DNA sequence variation. Future research should prioritize integrative multi-omics approaches and longitudinal study designs to better elucidate the dynamic interactions between genetic, epigenetic, and environmental factors underlying endurance performance. However, the implementation of comprehensive multi-omics frameworks in elite athlete populations remains challenging due to the rarity of these highly specialized cohorts and the limited sample sizes available for analysis. To overcome these constraints, future investigations should promote international collaborative consortia, harmonized phenotyping protocols, and shared athlete biobanks that facilitate data integration across research centers. The combination of deeply phenotyped longitudinal cohorts with advanced analytical approaches, including systems biology and machine learning methods, may further enhance the ability to extract biologically meaningful insights from relatively small populations. In addition, the integration of data from sub-elite and highly trained athletes may provide complementary evidence for validating molecular mechanisms identified in elite performers. Alongside these methodological advances, ethical considerations related to genetic data use, fairness, informed consent, and athlete privacy must remain a central priority. Such efforts will be essential for translating multi-omics discoveries into a better understanding of the molecular mechanisms underlying individual differences in endurance adaptation and performance. However, current evidence remains insufficient to support the use of genetic or multi-omics profiles for athlete selection or for designing training programs based on individual molecular profiles. Future research should first establish the reproducibility, predictive validity, and practical utility of these molecular signatures in well-characterized athletic populations.
